# Implications of multiple late gadolinium enhancement lesions on the frequency of left ventricular reverse remodeling and prognosis in patients with non‐ischemic cardiomyopathy

**DOI:** 10.1186/s12968-021-00734-3

**Published:** 2021-03-25

**Authors:** Shingo Ota, Makoto Orii, Tsuyoshi Nishiguchi, Mao Yokoyama, Ryoko Matsushita, Kazushi Takemoto, Takashi Tanimoto, Kumiko Hirata, Takeshi Hozumi, Takashi Akasaka

**Affiliations:** 1 Department of Cardiovascular Medicine, Wakayama Medical University, Wakayama, Japan; 2 Department of Radiology, Iwate Medical University, 1-1, Idaidori, Yahaba, 028-3695 Iwate, Japan; 3 Clinical Laboratory, Wakayama Medical University, Wakayama, Japan; 4 Department of Education, Division of medical science, Osaka Educational University, Osaka, Japan

**Keywords:** Multiple late gadolinium enhancement lesions, Cardiovascular magnetic resonance, Non‐ischemic cardiomyopathy, Reverse remodeling

## Abstract

**Background:**

Non-ischemic cardiomyopathy (NICM) is a heterogeneous disease, and its prognosis varies. Although late gadolinium enhancement (LGE)-cardiovascular magnetic resonance (CMR) demonstrates a linear pattern in the mid-wall of the septum or multiple LGE lesions in patients with NICM, the therapeutic response and prognosis of multiple LGE lesions have not been elucidated. This study aimed to investigate the frequency of left ventricular (LV) reverse remodeling (LVRR) and prognosis in patients with NICM who have multiple LGE lesions.

**Methods:**

This single-center retrospective study included 101 consecutive patients with NICM who were divided into 3 groups according to LGE-CMR results: patients without LGE (no LGE group = 48 patients), patients with a typical mid-wall LGE pattern (n = 29 patients), and patients with multiple LGE lesions (n = 24 patients). LVRR was defined as an increase in LV ejection fraction (LVEF) ≥ 10 % and a final value of LVEF > 35 %, which was accompanied by a decrease in LV end-systolic volume ≥ 15 % at 12-month follow-up using echocardiography. The frequency of composite cardiac events, defined as sudden cardiac death (SCD), aborted SCD (non-fatal ventricular fibrillation, sustained ventricular tachycardia, or adequate implantable cardioverter-defibrillator therapies), and heart failure death or hospitalization for worsening heart failure, were summarized and compared between the groups.

**Results:**

Among the 3 groups, the frequency of LVRR was significantly lower in the multiple lesions group than in the no LGE and mid-wall groups (no LGE vs. mid-wall vs. multiple lesions: 49 % vs. 52 % vs. 19 %, p = 0.03). There were 24 composite cardiac events among the patients: 2 in patients without LGE (hospitalization for worsening heart failure; 2), 7 in patients of the mid-wall group (SCD; 1, aborted SCD; 1 and hospitalization for worsening heart failure; 5), and 15 in patients of the multiple lesions group (SCD; 1, aborted SCD; 8 and hospitalization for worsening heart failure; 6). The multiple LGE lesions was an independent predictor of composite cardiac events (hazard ratio: 11.40 [95 % confidence intervals: 1.49−92.01], p = 0.020).

**Conclusions:**

Patients with multiple LGE lesions have a higher risk of cardiac events and poorer LVRR. The LGE pattern may be useful for an improved risk stratification in patients with NICM.

## Background

Non-ischemic cardiomyopathy (NICM) is characterized by a reduction in left ventricular (LV) systolic function in the absence of significant coronary artery disease. In particular, NICM with LV enlargement due to remodeling is called non-ischemic dilated cardiomyopathy (DCM) [1, 2]. NICM is a heterogeneous disease, and its response to therapy is varied. Therefore, precise phenotyping and personalized management are important to improve outcomes [3]. The evaluation of myocardial fibrosis is the key mechanism for distinguishing among phenotypes and predicting therapeutic reactivity in patients with NICM [4]. An endomyocardial biopsy is required for a conclusive diagnosis; however, the diagnostic value of endomyocardial biopsy is limited, and nonspecific myocardial fibrosis was observed in approximately 80 % of patients who underwent endomyocardial biopsy [5].

Areas of replacement fibrosis on histology are demonstrated as those of late gadolinium enhancement (LGE) on cardiovascular magnetic resonance (CMR). Approximately 30 % of patients with DCM have a characteristic linear pattern in the mid-wall of the septum on LGE-CMR [6, 7]. The mid-wall LGE, in addition to the LV ejection fraction (LVEF), provides predictive value for all-cause mortality, heart failure, and sudden cardiac death (SCD) [4].

CMR analysis also revealed that there are several patterns of LGE, such as multiple focal LGE, a combination of septal mid-wall and other types of LGE, and heterogeneous LGE in patients with NICM [8]. These patterns of LGE are frequently observed in acute and chronic myocarditis and cardiac sarcoidosis. However, patients with these LGE patterns sometimes do not meet the diagnostic criteria for chronic myocarditis and cardiac sarcoidosis because advanced imaging modalities can detect only active inflammation of the myocardium. Therefore, patients with LV systolic dysfunction who have multiple LGE lesions are usually diagnosed with NICM in daily clinical practice. In a previous study that reported the relationship between LGE patterns and prognosis, the “multiple pattern,”” which seems to be a combination of septal mid-wall LGE and other types of LGE, was found to be associated with the greatest SCD risk [8]; however, the association has not been completely elucidated. We hypothesized that multiple LGE lesions are associated with poorer LV reverse remodeling (LVRR) and cardiac events including deleterious ventricular arrhythmia and heart failure. To test the hypothesis, this study aimed to describe the frequency of LVRR and prognosis in patients with NICM who have multiple LGE lesions.

## Methods

### Study population

In this single-center, retrospective, observational study, consecutive patients with LV dilatation and systolic dysfunction, defined as LVEF < 50 % on echocardiography, who had symptoms of heart failure and abnormalities on electrocardiogram (ECG) or chest X-ray and who were referred to our institution between January 2010 and June 2015 were screened for the study registry. The diagnosis of NICM was made according to the World Health Organization/International Society and Federation of Cardiology criteria [9]. All patients were diagnosed with NICM within 1 month after CMR, and patients who had already been diagnosed and treated with NICM were not included. Exclusion criteria were as follows: ischemic heart disease defined as stenosis of > 50 % in a major coronary artery or clinical evidence of previous myocardial infarction; evidence of acute myocarditis or ongoing inflammatory myocardial disease; infiltrative cardiomyopathy; hypertrophic cardiomyopathy; arrhythmogenic right ventricular cardiomyopathy; or significant valve disease. We assessed the T2-weighted images in all patients who underwent CMR to exclude acute myocarditis, and additionally performed ^18^F-fluorodeoxyglucose positron emission tomography (^18^F-FDG PET) in patients with multiple LGE lesions to exclude cardiac sarcoidosis. We also excluded contraindications for LGE-CMR such as implanted metal in the body, claustrophobia, or significant renal dysfunction (estimated glomerular filtration rate (eGFR) < 30 ml/min/1.73 m^2^). Of 226 patients referred for possible enrollment in the study, 125 were excluded because of ischemic heart disease (n = 93), infiltrative cardiomyopathy (n = 18, number of patients newly diagnosed with cardiac sarcoidosis by ^18^F-FDG PET; 7 of 18 patients), significant valvular disease (n = 4), and significant renal dysfunction (n = 10). The final study population comprised 101 patients. Informed consent was obtained from each patient included in the registry, and informed written consent was obtained from all participants in this study. The study protocol was approved by the Ethics Committee of Wakayama Medical University.

### Clinical parameters

The assessed clinical parameters were gender, age, New York Heart Association classification (NYHA), risk factors (hypertension, diabetes), blood pressure, and medications at the beginning of the study. Brain natriuretic peptide (BNP), creatinine, and eGFR were obtained. We also assessed 12-month follow-up BNP values after standard medical therapy and analyzed the percent changes in BNP values (ΔBNP) from the beginning of the study to the 12-month follow-up. A 12-lead ECG was performed in all patients at the beginning of the study, and the presence or absence of atrial fibrillation and QRS duration were assessed.

### CMR protocol

CMR was performed during hospitalization for patients admitted due to the onset of heart failure and arrhythmia, and was performed electively for outpatients with stable condition. All CMR examinations were performed using a 1.5-T clinical CMR scanner (Intera Achieva; Philips Healthcare, Amsterdam, The Netherlands) equipped with a 32-element cardiac phased-array coil for signal reception, as previously described [10]. During the examination, the patients were continuously monitored using single-lead ECG and through repeated blood pressure measurements and pulse oximetry. With the patient in the supine position, contiguous short-axis cine images covering the LV from the base to the apex were acquired using a standard balanced steady-state free precession sequence. We then applied a breath-hold T2-weighted sequence with short-T1 inversion recovery for fat saturation. LGE-CMR imaging covering the whole ventricle was performed 10–15 min after intravenously injecting 0.1 mmol/kg gadolinium diethylenetriamine penta-acetic acid (Magnevist, Bayer Schering Pharma AG, Berlin, Germany). We used a 3D inversion-recovery turbo gradient echo sequence, and images were obtained during an end-expiratory breath-hold. Scan parameters were as follows: TR, 4.1 ms; TE, 1.25 ms; flip angle, 15°; FOV, 350 × 350 mm; partial echo; matrix, 224 × 256, and spatial resolution at 1.56 × 2.24 × 10 mm^3^ reconstructed to 0.68 × 0.68 × 5 mm^3^. Inversion time was adjusted to null the signal from viable myocardium [11].

### CMR analysis

**Fig. 1:**
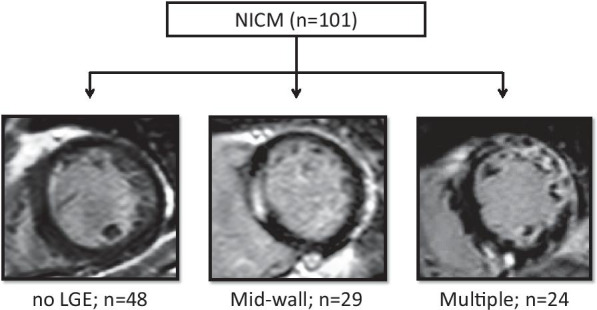
Late gadolinium enhancement patterns: all 101 patients with non-ischemic cardiomyopathy were divided into 3 groups: no LGE (48 patients), mid-wall (29 patients), and multiple lesions (24 patients). *NICM*  nonischemic cardiomyopathy, *LGE*  late gadolinium enhancement

All analyses were performed by consensus of independent blinded observers at an off-line workstation (ViewForum, Philips Healthcare). Normal myocardium was defined as normal regional wall thickening and the absence of any LGE on visual assessment. On LGE-CMR, we assessed the presence and pattern of LGE. According to a previous study [12], LGE was defined as an area with signal intensity of 5 SDs above the mean signal obtained in the normal myocardium on LGE images. We calculated LGE size by automatic summation of all slice volumes of the LGE area and expressed it as a percentage of LV volume. For patients with LGE, the LGE pattern was determined by the consensus of 2 cardiologists trained in CMR analysis (M.O. and T.T.) who were blinded to the clinical data. M.O. and T.T. had 15 and 20 years of CMR experience, respectively. The LGE pattern was defined visually as a linear pattern in the mid-wall of the septum or multiple LGE lesions. Multiple focal LGE, a combination of septal mid-wall and other types of LGE, and heterogeneous LGE were collectively categorized as multiple LGE lesions in our study. No isolated non-septal LGE was observed in this study population. If there was a difference in opinion about LGE pattern between two trained cardiologists, the LGE pattern was determined by discussion between them. All 101 patients with NICM were divided into 3 groups according to the findings of LGE-CMR: absence of LGE (no LGE group, n = 48), a linear pattern at the mid-wall of the septum (n = 29), and multiple LGE lesions (n = 24) (Fig. 1).

**Fig. 2:**
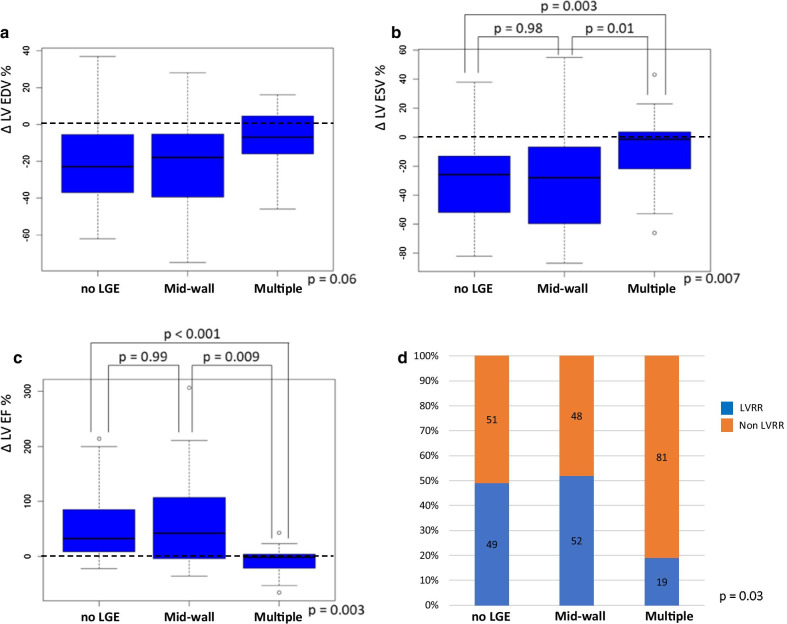
Changes in left ventricular end-diastolic volume (LVEDV), LV end-systolic volume (LVESV), and LV ejection fraction (LVEF), and frequency of reverse remodeling. **a** Percentage changes in LVEDV, **b** LVESV, and **c** LVEF among the 3 study groups (no LGE, a linear pattern at the mid-wall, and multiple lesions). **d** Comparison among the 3 groups focusing on the frequency of left ventricular reverse remodeling. *LVEDV*  left ventricular end-diastolic volume, *LVEF*  left ventricular ejection fraction, *LVESV*  left ventricular end-systolic volume, *LGE*  late gadolinium enhancement, *LVRR*  left ventricular reverse remodeling

### LVRR

Transthoracic echocardiographic measurements (Vivid E9, General Electric Healthcare, Waukesha, Wisconsin, USA) were performed at the time of diagnosis of NICM (baseline) and 12 months after standard medical therapy. LV end-diastolic volume (LVEDV), LV end-systolic volume (LVESV), and LVEF were calculated using the modified biplane Simpson method [13]. An analysis was performed based on percent changes in LVEDV (ΔLVEDV), LVESV (ΔLVESV), and LVEF (ΔLVEF) from baseline to the 12-month follow-up echocardiographic examination. LVRR was defined as an increase in LVEF ≥ 10 % and a final value of LVEF > 35 %, which was accompanied by a decrease in LVESV ≥ 15 % at 12-month follow-up [14, 15]. LVEDV, LVESV and LVEF were measured by 2 experienced sonographers (K.T. and Y. M.) who were blinded to the clinical data. We evaluated the intra- and inter-rater reliability of the echocardiographic measurements using intraclass correlation coefficient (ICC).

### Follow‐up and clinical events

We investigated clinical events during the follow-up period from the performance of CMR to the end of June 2019. Patient follow-up was conducted via telephone interview with patients, and medical records review. Information on composite cardiac events, defined as SCD, aborted SCD (non-fatal ventricular fibrillation, sustained ventricular tachycardia, or adequate implantable cardioverter-defibrillator (ICD) therapies), and heart failure death or hospitalization for worsening heart failure were collected.

### Statistical analysis

All statistical analyses were performed using JMP (version 14, SAS Institute Inc., Cary, North Carolina, USA). Categorical variables are presented as frequency counts and percentages and comparisons were performed using Fisher’s exact test. Continuous variables are presented as mean ± standard deviation and compared using the Kruskal-Wallis test. Steel-Dwass post hoc analysis was used to compare the 12-month follow-up BNP values and percent changes in BNP values, LVEF, and LVESV between each group of patients. Kaplan-Meier curves were used to visualize the composite cardiac event-free cumulative survival of patients among the 3 groups. Log-rank tests were performed to compare survival curves. The hazard ratio (HR) was calculated using a Cox regression model with 95 % confidence intervals (CIs). Model 1 included the baseline characteristics with p < 0.05 in the univariate analysis. Model 2 included all 12-month follow-up parameters, such as 12-month follow-up BNP, LVEDV, LVESV, LVEF, and non-LVRR. All tests were two-sided, and p < 0.05 was considered statistically significant.

## Results

### Baseline characteristics

Baseline clinical characteristics among the 3 groups are listed in Table 1.

**Table 1:** Baseline patient characteristics

	No LGE group n = 48	Mid-wall LGE group n = 29	Multiple LGE lesions group n = 24	p-value
Male	31 (65)	26 (89)	18 (75)	0.06
Age, years	60.2 ± 12.8	63.4 ± 13.4	60.1 ± 10.7	0.36
NYHA				
I	7 (15)	1 (3)	6 (25)	0.06
II	15 (31)	5 (17)	7 (29)	
III	15 (31)	19 (66)	8 (33)	
IV	11 (23)	4 (14)	3 (13)	
NYHA ≥ III	26 (54)	23 (79)	11 (46)	0.02
BNP, pg/dL	603 ± 567	898 ± 647	461 ± 974	< 0.001
Hypertension	23 (48)	16 (55)	10 (43)	0.71
Diabetes	11 (23)	6 (21)	4 (17)	0.95
Creatinine, mg/dL	0.8 ± 0.2	1.0 ± 0.3	0.8 ± 0.2	0.02
eGFR, ml/min/1.73 m^2^	73.0 ± 25.1	60.5 ± 19.1	70.0 ± 17.8	0.08
Systolic BP, mmHg	130 ± 25	127 ± 23	125 ± 21	0.76
Diastolic BP, mmHg	80 ± 18	84 ± 20	76 ± 15	0.39
Atrial fibrillation	3 (6)	3 (10)	3 (13)	0.60
QRS duration, ms	111 ± 22	109 ± 25	120 ± 25	0.21
LVEDV, mL	1780 ± 45	196 ± 69	168 ± 38	0.16
LVESV, mL	126 ± 44	144 ± 64	106 ± 29	0.02
LVEF, %	31 ± 10	29 ± 10	37 ± 8	0.007
LGE size, %	0	3.1 ± 3.7	22.0 ± 11.7	< 0.001
Medications				
Furosemide	28 (58)	25 (86)	13 (57)	0.02
ACE-i/ARB	44 (92)	23 (79)	18 (78)	0.18
Spironolactone	15 (31)	15 (52)	8 (35)	0.20
Beta-blocker	43 (90)	28 (97)	20 (87)	0.39
Amiodarone	6 (13)	8 (28)	8 (35)	0.07

Values are expressed as mean ± SD or n (%). *ACE-i*  angiotensin converting enzyme inhibitor, *ARB*  angiotensin receptor blocker, *BNP*  brain natriuretic peptide, *BP*  blood pressure, *eGFR*  estimated glomerular filtration rate, *LGE*  late gadolinium enhancement, *LVEDV*  left ventricular end-diastolic volume, *LVEF*  left ventricular ejection fraction, *LVESV*  left ventricular end-systolic volume, *NYHA*  New York Heart Association classification

There were no significant differences among the groups with regard to gender, age, hypertension, diabetes, eGFR, blood pressure, atrial fibrillation, QRS duration, medications at the beginning of the study, and LVEDV. The number of NYHA ≥ III was significantly higher in the mid-wall LGE group than in the no LGE and multiple lesion groups (no LGE vs. mid-wall vs. multiple lesions: 54 % vs. 79 % vs. 46 %, p = 0.02). Serum BNP and creatinine levels were significantly higher in the mid-wall group than in the no LGE and multiple lesion groups (no LGE vs. mid-wall vs. multiple lesions: 603 ± 567 pg/dL vs. 898 ± 647 pg/dL vs. 461 ± 974 pg/dL, p < 0.001, and 0.8 ± 0.2 mg/dL vs. 1.0 ± 0.3 mg/dL vs. 0.8 ± 0.2 mg/dL, p = 0.02, respectively). The mean LVESV at baseline, acquired via echocardiography, was significantly higher in the mid-wall group than in the no LGE and multiple lesion groups (no LGE vs. mid-wall vs. multiple lesions: 126 ± 44 mL vs. 144 ± 64 mL vs. 106 ± 29 mL, p = 0.02). The mean LVEF at baseline, acquired via echocardiography, was significantly lower in the mid-wall group than in the no LGE and multiple lesion groups (no LGE vs. mid-wall vs. multiple lesions: 31.1 ± 10.0 % vs. 28.6 ± 10.3 % vs. 36.7 ± 8.2 %, p = 0.007). The LGE size was significantly larger in the multiple lesion group than in the no LGE and mid-wall groups (no LGE vs. mid-wall vs. multiple lesions: 0.0 % vs. 3.1 ± 3.7 % vs. 22.0 ± 11.7 %, p < 0.001).

### Follow‐up echocardiography and BNP values

**Fig. 3:**
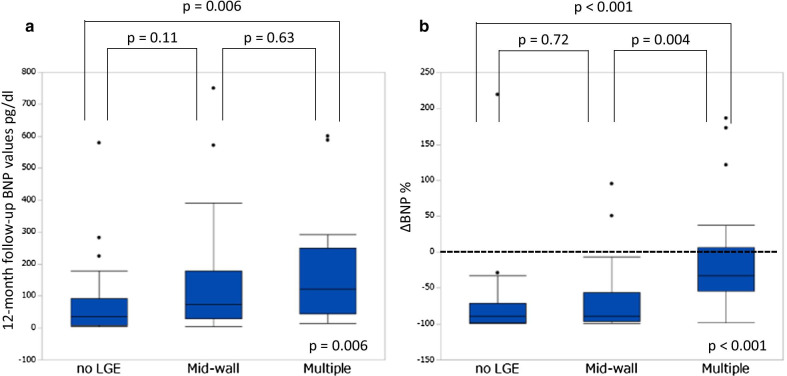
12-Month follow-up brain natriuretic peptide (BNP) values, and changes in BNP values. **a** The 12-month follow-up BNP values, and **b** percent changes in BNP values among the 3 study groups (no LGE, linear pattern at the mid-wall, and multiple lesions). *BNP*  brain natriuretic peptide, *LGE*  late gadolinium enhancement

The 12-month follow-up echocardiography was performed in 41 (85 %) of 48 patients without LGE, 27 (93 %) of 29 patients with mid-wall, and 21 (88 %) of 24 patients with multiple lesions. The intra- and inter-rater reliabilities of LVEDV, LVESV, and LVEF were ICC (1,2) = 0.977 and ICC (2,2) = 0.976, ICC (1,2) = 0.980 and ICC (2,2) = 0.979, and ICC (1,2) = 0.961 and ICC (2,2) = 0.961, respectively. ΔLVEDV, ΔLVESV, and ΔLVEF among the 3 groups at the end of the 12-month follow-up are shown in Fig. 2a–c. There were no significant differences among the 3 groups in ΔLVEDV (no LGE vs. mid-wall vs. multiple lesions: –20.9 ± 22.3 % vs. –22.2 ± 24.5 % vs. –9.6 ± 17.2 %, p = 0.05). The percent increase in LVEF was significantly lower in the multiple lesion group than in the no LGE and mid-wall groups (no LGE vs. mid-wall vs. multiple lesions: 48.9 ± 56.8 % vs. 62.2 ± 81.3 % vs. 5.1 ± 29.4 %, p = 0.003). The percent reduction in LVESV was significantly lower in the multiple lesion group than in the no LGE and mid-wall groups (no LGE vs. mid-wall vs. multiple lesions: –30.1 ± 29.5 % vs. –30.3 ± 33.9 % vs. –7.7 ± 25.9 %, p = 0.007). Figure 2d shows a comparison among the 3 groups focusing on the frequency of LVRR at 12 months. Among the 3 groups, the frequency of LVRR was significantly lower in the multiple lesion group than in the no LGE and mid-wall groups (no LGE vs. mid-wall vs. multiple lesions: 49 % vs. 52 % vs. 19 %, p = 0.03). The 12-month follow up BNP values were significantly lower in the no LGE group (no LGE vs. mid-wall vs. multiple lesions: 73 ± 111pg/dl vs. 153 ± 201pg/dl vs. 165 ± 168pg/dl, p = 0.006) (Fig. 3a). The percent decrease in BNP values was significantly lower in the multiple lesion group than in the no LGE and mid-wall groups (no LGE vs. mid-wall vs. multiple lesions: –72.9 ± 54.8 % vs. –64.2 ± 52.4 % vs. –8.5 ± 79.4 %, p < 0.001) (Fig. 3b). 

### Follow‐up and clinical events

**Fig. 4:**
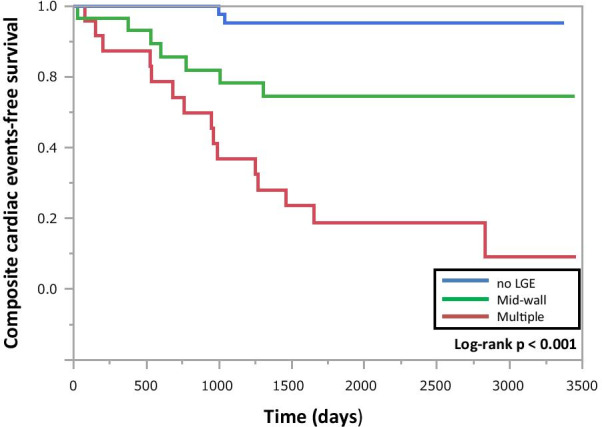
Composite cardiac events. Kaplan–Meier survival curves according to the composite cardiac events. *LGE * late gadolinium enhancement

Follow-up data were obtained for all 101 patients. They were followed up for a median of 1972 (interquartile range, 1005–2541) days. Nineteen patients received ICD implantation during the follow-up period (4 patients without LGE, 5 in the mid-wall group, and 10 in the multiple lesions group). Five patients received adequate ICD therapies (no patient without LGE, 1 patient in the mid-wall group, and 4 patients in the multiple lesion group). There were 24 composite cardiac events among the patients: 2 in patients without LGE (hospitalization for worsening heart failure; 2), 7 in patients of the mid-wall LGE group (SCD; 1, aborted SCD; 1 and hospitalization for worsening heart failure; 5), and 15 in patients of the multiple lesion group (SCD; 1, aborted SCD; 8 and hospitalization for worsening heart failure; 6). The Kaplan-Meier curves are shown in Fig. 4. The composite cardiac events were significantly more frequent in patients with multiple lesions than in those with mid-wall and without LGE (p < 0.001). The multiple LGE lesions, baseline BNP, and creatinine were independent predictors of composite cardiac events (HR: 11.4 [95 % CIs: 1.49−92.0], p = 0.020, HR: 0.997 [95 % CIs: 0.994−0.999], p = 0.017 and HR: 81 [95 % CIs: 6 −1122], p = 0.001, respectively) (Table 2**)**. Moreover, 12-month follow-up BNP and non-LVRR were independent predictors of composite cardiac events (HR: 1.003 [95 % CIs: 1.001−1.006], p = 0.010, HR: 5.85 [95 % CIs: 1.2−35.2], p = 0.030, respectively) **(**Table 3**)**.

**Table 2:** Prediction of composite cardiac events in patients with NICM (model 1)

	HR (95 % CIs)	p-value
Multiple LGE lesions	11.4 (1.5–92.0)	0.020
NYHA ≥ III	0.77 (0.16–4.2)	0.756
BNP	0.997 (0.994–0.999)	0.017
Creatinine	81 (6–1122)	0.001
Furosemide	7.4 (0.9–68.3)	0.067
LGE size	1.9 (0.1–34.7)	0.658
LVESV	0.991 (0.970–1.013)	0.429
LVEF	0.995 (0.887–1.116)	0.925

*BNP* brain natriuretic peptide, *CIs* confidence intervals, *HR* hazard ratio, *LGE* late gadolinium enhancement, *LVEF* left ventricular ejection fraction, *LVESV* left ventricular end-systolic volume, *NICM* non-ischemic cardiomyopathy, *NYHA* New York Heart Association classification

**Table 3:** Prediction of composite cardiac events in patients with NICM (model 2)

	HR (95 % CIs)	p-value
12-Month follow-up BNP	1.003 (1.001–1.006)	0.010
12-Month follow-up LVEDV	1.090 (0.985–1.236)	0.142
12-Month follow-up LVESV	0.894 (0.735–1.049)	0.220
12-Month follow-up LVEF	0.858 (0.617–1.123)	0.321
Non LVRR	5.85 (1.19–35.23)	0.030

*BNP* brain natriuretic peptide, *CIs* confidence intervals, *HR* hazard ratio, *LVEDV* left ventricular end-diastolic volume, *LVEF* left ventricular ejection fraction, *LVESV* left ventricular end-systolic volume, *LVRR* left ventricular reverse remodeling, *NICM* non-ischemic cardiomyopathy

## Discussion

To the best of our knowledge, we demonstrated for the first time the frequency of LVRR and prognosis in patients with NICM who have multiple LGE lesions. The major findings of this study are (1) The multiple LGE lesions show poorer LVRR, (2) The composite cardiac events are significantly more frequent in patients with multiple LGE lesions, and the presence of multiple LGE lesions is an independent predictor of the composite cardiac events.

Halliday et al. reported the relationship between the pattern of LGE and outcomes in patients with DCM [8]. As the number of patients in our study was lower than that in their study, we could not classify the various LGE patterns as they did. Therefore, atypical LGE patterns, such as multiple focal LGE, a combination of septal mid-wall and other types of LGE, and heterogeneous LGE were collectively categorized as multiple LGE lesions in our study. Conversely, Halliday et al. described a combination of septal mid-wall and other types of LGE as “multiple pattern.” Thus, the definition of multiple LGE lesions in this study does not completely correspond to that provided in the study by Halliday et al., and this may be one of the reasons the percentage of patients with this pattern appears to be greater in our study than previously described. Multiple LGE lesions were frequently observed in patients with acute myocarditis and cardiac sarcoidosis; however, we carefully excluded these diseases by confirming both normal intensity on T2-weighted CMR and negative ^18^F−FDG PET uptake in this study. Moreover, in our study, endomyocardial biopsy was performed in 38 of 101 patients (no LGE group, 14; mid-wall group, 9; multiple lesions group, 15). Nonspecific pathological findings were observed in all 38 patients who underwent endomyocardial biopsy, which were described as “compatible with dilated cardiomyopathy” in the pathology reports.

LVRR is related to favorable prognosis in DCM patients [16, 17]. Before the era of beta-blockers, one study reported an LVRR incidence of only 27 % [18]. Over time, advances in heart failure pharmacotherapy have increased the incidence of LVRR in patients with DCM. Recent, results from the Intervention in Myocarditis and Acute Cardiomyopathy trials revealed the incidence of LVRR to be 70 % at 6 months and 56 % at 12 months [19, 20]. The prevalence of LVRR at 12 months in the present study was 43 %.

In contrast, predicting who can achieve LVRR by standard medical therapy has been difficult. Many studies have demonstrated that a linear mid-wall LGE pattern is associated with an increased risk of death, heart failure events, and arrhythmic events [4], and patients with a linear mid-wall LGE pattern are less likely to exhibit LVRR [14]. However, in the present study, the frequency of LVRR was significantly lower in patients with multiple LGE lesions than in those without LGE and in those with mid-wall LGE pattern. Although the serum BNP level and LVESV were significantly higher and LVEF was significantly lower at baseline in patients with a linear mid-wall LGE, the frequency of LVRR in patients with a linear mid-wall LGE was similar that in patients without LGE. These findings suggest that the LGE pattern, not the presence or absence of typical mid-wall LGE pattern, may be useful in predicting LVRR in patients with NICM.

In our study, we did not use CMR to assess LVRR. It is well known that, in comparison with CMR, LV volumes may be significantly underestimated in patients with normal and decreased LV function when assessed through 2D echocardiography owing to the poor image quality, apical foreshortening and geometrical assumptions [21–23]. CMR has proved to be a more accurate non-invasive tool than 2D echocardiography for measuring LV volumes and function. However, as this was a retrospective observational study, we could not perform a follow-up CMR in all patients. Compared to the number of patients who were available for echocardiography (88 %), the number of patients who were available for follow-up CMR was only 6 % of the study population; therefore, we assessed LVRR using echocardiography in this study. There were high intra- and inter-rater reliabilities in the measurements of LV volumes and function. Based on these results, it was reasonable to assess LVRR using echocardiography in this study. The number of patients who received a follow-up echocardiography at 12 months was relatively low. All patients who did not receive the 12-month follow-up echocardiography were alive at the time of follow-up, they could not visit our hospital for geographical reasons. A 12-month follow-up echocardiography was performed in 41 (85 %) of 48 patients without LGE, 27 (93 %) of 29 patients with mid-wall LGE, and 21 (88 %) of 24 patients with multiple LGE lesions. Although no LGE group had the lowest proportion of patients receiving follow-up echocardiography among the 3 groups, there were almost achievements of LVRR and few cardiac composite events in patients without LGE. Therefore, a relatively low echocardiographic follow-up rate seems to have little impact on this result.

In this study, the patients with multiple LGE lesions had a higher risk of composite cardiac events including deleterious ventricular arrhythmia and heart failure. There is emerging evidence suggesting that replacement fibrosis forms the substrate for ventricular arrhythmias owing to scar-related reentry [24, 25]. In multiple LGE lesions, the areas of a scar with the greatest heterogeneity may cause the largest variation in conduction velocities and have the greatest chance of resulting in re-entrant arrhythmia. Moreover, there are poorer rates of LVRR in patients with multiple LGE lesions, this phenomenon may contribute to the onset of heart failure and worsening. Therefore, these may be the mechanisms underlying composite cardiac events in patients with multiple LGE lesions. We also showed that the multiple LGE lesions were associated with a larger extent of LGE. A large extent of LGE has been reported as a strong predictor of deleterious ventricular arrhythmia in patients with DCM [26]; however, our study showed that the multiple LGE lesions, not the extent of LGE, was an independent predictor. The quantitative evaluation of LGE is important; however, this study suggested that visual evaluation of features such as the LGE pattern may be useful in predicting the prognosis in patients with NICM. Moreover, the percent decrease in BNP values was significantly lower in patients with multiple LGE lesions, and this result may support our major findings that the multiple LGE lesions show poorer LVRR and more frequent adverse cardiac events. The 12-month follow-up BNP and the frequency of non-LVRR were significantly higher in the multiple LGE lesions group; the 12-month follow-up BNP and non-LVRR were independent predictors of composite cardiac events that were compatible with poorer prognoses in patients with multiple LGE lesions.

### Limitations

This study had several limitations. First, the study was a single-center retrospective observational study, which limited the ability to determine cause and effect. In the future, a larger multicenter prospective study is needed to incorporate these findings into the clinical management of patients with NICM. Second, although we included patients with normal intensity on T2-weighted CMR and negative ^18^F-FDG PET uptake in this study, chronic myocarditis and cardiac sarcoid were not completely excluded. Furthermore, in this study population, T2 weighted images were obtained from 87 hospitalized patients and 14 elective outpatients. Therefore, we may have lost the timing of detection of acute myocardial inflammation in 14 elective outpatients. Additionally, there was a high prevalence of hypertension in this cohort. Therefore, it is possible that some patients with hypertensive cardiomyopathy were included, which might have affected the frequency of LVRR, especially in patients without LGE. Third, we did not assess the troponin values at baseline. Measurement of troponin values is useful not only for the diagnosis of acute myocarditis but also for prognostic prediction in patients with NICM. Further studies that include assessment of troponin values will be needed. Forth, follow-up CMR images were not available, and therefore, we did not assess the change in LGE patterns. A previous study reported that LGE disappeared in 35 % of patients with DCM who had LGE at baseline and that 20 % of patients with DCM who did not have LGE at baseline developed a new LGE lesion [15]. The detailed mechanism of the disappearance of LGE is unknown. LGE can demonstrate not only replacement fibrosis but also the presence of edema and inflammatory cell infiltration. Thus, among the patients in whom LGE disappeared at follow-up, patients with inflammatory diseases such as acute myocarditis might masquerade as those with DCM. All patients in our study were negative for the T2-weighted CMR, therefore, it was unlikely that LGE had disappeared. The study performing a follow-up CMR is needed to evaluate the change of LGE. Fifth, the information regarding medical therapy was given only at the beginning of the study. Physicians tried to continue these medications until the end of the study, but the detailed changes in medications, such as an increase in the dosage of beta-blockers, were not followed. The changes in medications during the follow-up period might have influenced the LVRR and prognosis among the 3 groups. Finally, the left anterior descending artery gives a variable number of septal perforator branches. A large first septal perforator branch may mimic a linear mid-wall LGE at the base of the heart [27]. We did not have any computed tomography angiographies to ascertain whether the septal perforator branches were not erroneously included as a linear mid-wall LGE.

## Conclusions

Patients with NICM who have multiple LGE lesions have a higher risk of cardiac events and poorer LVRR despite receiving standard medical therapy. The LGE pattern may be useful for an improved risk stratification in patients with NICM.

## Data Availability

The dataset analyzed during the current study are available from the corresponding author on reasonable request.

## Abbreviations

ACE-i: Angiotensin converting enzyme inhibitor
ARB: Angiotensin receptor blocker
BNP: Brain natriuretic peptide
BP: Blood pressure
CI: Confidence intervals
DCM: Non-ischemic dilated cardiomyopathy
ECG: Electrocardiogram
eGFR: Estimated glomerular filtration rate
^18^F-FDG PET: ^18^F-fluorodeoxyglucose positron emission tomography
HR: Hazard ratio
ICD: Implanted cardiodefibrillator
LGE: Late gadolinium enhancement
LV: Left ventricle/left ventricula
LVEDV: Left ventricular end-diastolic volume
LVEF: Left ventricular ejection fraction
LVESV: Left ventricular end-systolic volume
LVRR: Left ventricular reverse remodeling
NICM: Non-ischemic cardiomyopathy
NYHA: New York Heart Association classification
SCD: Sudden cardiac death
